# Comparative in vitro analysis of inhibition of rhinovirus and influenza virus replication by mucoactive secretolytic agents and plant extracts

**DOI:** 10.1186/s12906-020-03173-2

**Published:** 2020-12-23

**Authors:** Christin Walther, Kristin Döring, Michaela Schmidtke

**Affiliations:** Department Medical Microbiology, Section Experimental Virology, Jena University Hospital, Hans-Knöll-Str. 2, D-07745 Jena, Germany

**Keywords:** Acute respiratory infection, Virus, Natural products, Over-the-counter medication, Ambroxol, Bromhexine, N-acetyl cysteine, Thyme, Pelargonium, Antiviral, Mechanism

## Abstract

**Background:**

Rhinoviruses and influenza viruses cause millions of acute respiratory infections annually. Symptoms of mild acute respiratory infections are commonly treated with over-the-counter products like ambroxol, bromhexine, and N-acetyl cysteine, as well as of thyme and pelargonium extracts today. Because the direct antiviral activity of these over-the-counter products has not been studied in a systematic way, the current study aimed to compare their inhibitory effect against rhinovirus and influenza virus replication in an in vitro setting.

**Methods:**

The cytotoxicity of ambroxol, bromhexine, and N-acetyl cysteine, as well as of thyme and pelargonium extracts was analyzed in Madin Darby canine kidney (MDCK) and HeLa Ohio cells. The antiviral effect of these over-the-counter products was compared by analyzing the dose-dependent inhibition (i) of rhinovirus A2- and B14-induced cytopathic effect in HeLa Ohio cells and (ii) of influenza virus A/Hong Kong/68 (subtype H3N2)- and A/Jena/8178/09 (subtype H1N1, pandemic)-induced cytopathic effect in MDCK cells at non-cytotoxic concentrations. To get insights into the mechanism of action of pelargonium extract against influenza virus, we performed time-of-addition assays as well as hemagglutination and neuraminidase inhibition assays.

**Results:**

N-acetyl cysteine, thyme and pelargonium extract showed no or only marginal cytotoxicity in MDCK and HeLa Ohio cells in the tested concentration range. The 50% cytotoxic concentration of ambroxol and bromhexine was 51.85 and 61.24 μM, respectively. No anti-rhinoviral activity was detected at non-cytotoxic concentrations in this in vitro study setting. Ambroxol, bromhexine, and N-acetyl cysteine inhibited the influenza virus-induced cytopathic effect in MDCK cells no or less than 50%. In contrast, a dose-dependent anti-influenza virus activity of thyme and pelargonium extracts was demonstrated. The time-of addition assays revealed an inhibition of early and late steps of influenza virus replication by pelargonium extract whereas zanamivir acted on late steps only. The proven block of viral neuraminidase activity might explain the inhibition of influenza virus replication when added after viral adsorption.

**Conclusion:**

The study results indicate a distinct inhibition of influenza A virus replication by thyme and pelargonium extract which might contribute to the beneficial effects of these plant extracts on acute respiratory infections symptoms.

## Background

Acute respiratory infections affect the upper and/or lower respiratory tract and account for the majority of infectious diseases worldwide. Due to the high number of hours of school and work absence, primary care, hospitalization, medications etc. acute respiratory infections have a huge medical and economic impact. Severe infections of the lower respiratory tract even represent the fourth most common reason of death worldwide [1].

Acute respiratory infections are commonly caused by viruses [2] whereat new viruses can emerge and cause pandemics as seen for example for influenza A viruses of subtype H1N1 in 2009 and for coronaviruses in 2019 [3]. In particular, rhinoviruses, influenza viruses, and coronaviruses are frequently diagnosed in acute respiratory infections with mild as well as severe respiratory symptoms, including acute lung injury [4, 5]. Annual influenza epidemics are estimated to result in about 3 to 5 million cases of severe illness, and about 290,000 to 650,000 respiratory deaths [6]. Measures of prevention and treatment of rhinovirus and coronavirus infections do not exist, albeit several virus proteins e. g. viral capsid, protease, and polymerase proteins have been identified as valid targets of inhibitors [7–9]. Even though influenza vaccines do exist, they are suboptimal applied [10–13]. The arsenal of drugs for treatment of influenza is limited. It includes M2 ion channel blockers (amantadine and rimantadine), neuraminidase inhibitors (oseltamivir, zanamivir, laninamivir, and peramivir), and polymerase inhibitors (favipiravir and baloxavir) today [14, 15]. Moreover, there is a permanent risk of emergence of drug-resistant influenza viruses due to the high genetic variability based on point mutations and gene reassortment [16]. For example, the two M2 ion channel blockers are not recommended for use in monotherapy because the circulating influenza A viruses are resistant today [6]. Of note, ion channel blockers do generally not act against influenza B viruses due to structural differences in the viral target [17]. According to recommendations of the World Health Organization, “Patients with severe or progressive clinical illness associated with suspected or confirmed influenza virus infection (i.e. clinical syndromes of pneumonia, sepsis or exacerbation of chronic underling diseases) should be treated with antiviral drugs as soon as possible.” [6]. Ideally treatment should be started within 48 h following symptom onset to maximize therapeutic benefits but, drug administration should also be considered in patients presenting later in the course of illness [6].

The World Health Organization further recommends that influenza patients who are not in a high-risk group “should be managed with symptomatic treatment and are advised, if symptomatic, to stay home in order to minimize the risk of infecting others in the community. Treatment focuses on relieving symptoms of influenza such as fever.” [6]. In fact, the management of upper and lower respiratory tract infections often relies on the use of self-medicated over-the-counter medicines [18]. According to this European study analyzing the medication use in primary care patients with lower respiratory tract infections, 55.4% of patients self-medicated before consultation and 21.5% after consultation. Mucolytic agents were amongst the most frequently used self-medications. Mucolytic agents like ambroxol (a metabolite of bromhexine), bromhexine, and N-acetyl cysteine are also widely used in the therapy of chronic obstructive pulmonary disease (reduction of frequency and duration of exacerbations), bronchitis and sinusitis [19]. The mucolytic, anti-inflammatory and anti-oxidant properties of mucolytic agents have been shown to contribute to symptom relief [19, 20] but further research is needed to prove their efficacy [18, 19]. Furthermore, thyme and pelargonium extract are frequently used for symptomatic treatment of acute respiratory infections [21–23].

The antiviral potential of over-the-counter medicines has been studied sporadically. Few studies were performed with ambroxol [24, 25], N-acetyl cysteine [26–29], and pelargonium extracts [30–32] in vitro and/or in vivo. The results are hard to compare because, in part, very high agent concentrations, different experimental conditions (e. g. cell lines or primary cells, different treatment schedules, infections dose, and controls), and study parameters (e.g. lethality and time of symptom release) were applied. Albeit the study results show beneficial treatment effects in vitro and in vivo, it is difficult to draw conclusions on the effects of most of these self-medications on rhinovirus and/or anti-influenza virus replication. Although in vitro experiments cannot mimic the multifactorial and very complex in vivo situation and have only a limited predictive value for the clinical setting, antiviral effects shown in vitro can help to explain treatment success seen in vivo or in a clinical setting.

In this study, we aimed to comparatively analyze the effect of ambroxol, bromhexine, N-acetylcysteine, thyme, and pelargonium extract on the replication of rhinoviruses and influenza A viruses at non-cytotoxic micromolar concentrations in vitro. The well-known inhibitors pleconaril and zanamivir were used to validate the antiviral assays with rhinoviruses and influenza A viruses, respectively. To ensure the selectivity of the antiviral effect, we analyzed the cytotoxicity of all extracts and compounds in the cell lines used for antiviral studies. Moreover, we performed time-of-addition studies with the anti-influenza virus-active pelargonium extract to get insights into its mechanism of action.

## Methods

### Reference compounds and test items

As there are no drugs to treat rhinovirus infections, we applied pleconaril, a capsid-binding inhibitor with well-known antirhinoviral activity to validate the antiviral assays. The sensitivity of rhinoviruses to pleconaril has been determined in previous studies [33, 34]. For influenza A viruses the drug zanamivir (GlaxoSmithKline) was used as reference compound. Zanamivir is an approved, direct anti-influenza virus-acting drug targeting the viral neuraminidase [35]. The zanamivir sensitivity of the used influenza A viruses was known [36, 37]. Stock solutions of pleconaril and zanamivir (10,000 μM) were prepared in dimethyl sulfoxide and bi-distilled water, respectively. The highest dimethyl sulfoxide concentration in the test was 0.05%.

The cytotoxic and antiviral activities of ambroxol and bromhexine hydrochloride (Boehringer Ingelheim Pharma GmbH & Co KG, Ingelheim, Germany), N-acetylcysteine (SIGMA-Aldrich Chemie GmbH, Schnelldorf, Germany), fluid thyme extract (R&R Extrakte GmbH; Cologne, Germany), pelargonium extract (A. Nattermann & Cie. GmbH; Cologne, Germany) were compared in this study. According to the supplier information, the fluid thyme extract consists 31.2% ethanol and 0.06% (v/v) thymol (phenol calculated as thymol) and pelargonium root powder extract 30.2% (m/m) polyphenols and 0.5% (m/m) umckalin. The thyme extract was used as provided. Stock solutions of the other test items were prepared in dimethyl sulfoxide in the following concentrations: 10,000 μg/mL of pelargonium extract or 10,000 μM for ambroxol, bromhexine, and N-acetylcysteine.

Working solutions of the reference compounds and test items were done in the test medium described in the following paragraph.

### Cell lines and virus strains

HeLa Ohio (human cervix carcinoma; Flow Labs; USA) and Madin Darby canine Kidney (MDCK; Friedrich Löffler Institute; Germany) cells allow the determination of cytotoxicity as well as antiviral activity of compounds and extracts against rhinoviruses and influenza A viruses, respectively [38]. The growth medium for propagation of HeLa Ohio cells contained Eagle’s minimal essential medium, supplemented with 5% fetal calf serum, 2 mM L-glutamine, and 1% nonessential amino acids. Eagle’s minimal essential medium with 10% fetal calf serum, 2 mM L-glutamine, and 1% nonessential amino acids was used to propagate MDCK cells.

The Eagle’s minimal essential medium for antirhinoviral tests (test medium) in HeLa Ohio cells was supplemented with 2% fetal calf serum only. The antiviral tests with influenza A viruses were performed in MDCK cells with Eagle’s minimal essential medium supplemented with 2.3% sodium bicarbonate, 2 μg/mL trypsin, 2 mM L-glutamine, and 1% nonessential amino acids (test medium).

Viruses included in this study were rhinovirus A2 (Institute of Biochemistry, University, Vienna, AUT), rhinovirus B14 (Charité, Berlin, Germany), the H3N2 influenza virus A/Hong Kong/68 (Schaper and Brümmer, Salzgitter, Germany), and the A(H1N1)pdm09 influenza virus A/Jena/8178/09 (isolated and kindly provided by Andy Krumbholz [37]). Rhinoviruses and influenza A viruses were grown and titrated in HeLa Ohio and MDCK cells, respectively. The determined virus titers of rhinovirus A2, rhinovirus B14, influenza virus A/Hong Kong/68, and influenza virus A/Jena/8178/09 were 6.3 × 10^6^ TCID_50_/mL, 2.0 × 10^6^ TCID_50_/mL, 2.0 × 10^7^ TCID_50_/mL, and 6.3 × 10^7^ TCID_50_/mL, respectively. Aliquots of the virus working passages were stored at − 80 °C until use.

### Cytotoxicity determination

HeLa Ohio and MDCK cells were seeded at 1.6 × 10^4^ and 2.3 × 10^4^ cells/well in 100 μL growth medium in 96 well flat-bottomed microtiter plates, respectively. The cytotoxicity of the test compounds and extracts was determined on two-day-old confluent cell monolayers grown in the internal 60 wells of a microtiter plate (5% carbon dioxide, 37 °C). After removal of the growth medium, 50 μL of test medium and eight half-log dilutions of the reference compounds pleconaril or zanamivir, or eight half-log dilutions of test items in test medium (each concentration in duplicates) were added. The concentration range of reference compounds applied to cytotoxicity assays was 0.0316–100 μM. The concentration range of test items was 0.0003–0.5% v/v for thyme extract, 0.0316–100 μg/mL for pelargonium extract, and 0.0316–100 μM for ambroxol, bromhexine, and N-acetylcysteine. Six cell control wells were incubated with 100 μL test medium. After 72 h of incubation at 37 °C in 5% carbon dioxide atmosphere, the Dynex Immuno Assay System (Guernsey, UK), developed for automated ELISA techniques, was applied to gently wash, stain, measure, and analyze the viability of the cell monolayers based on optical density determination [39]. After aspiration of supernatant, cell monolayers were washed three times with 300 μL physiological phosphate-buffered saline to remove death cells. The remaining cells were fixed and stained with 50 μL of 0.07% crystal violet (w/v), 20% ethanol, 3% formalin solution in water for 10 min. Six washings with 300 μL of water followed to remove excess stain. Before measuring the optical density, we solubilized the crystal violet by adding 100 μL lysis buffer (0.898 g of sodium citrate and 1.25 mL of 1 N HCl in 98.05 mL 47.5% ethanol) for 20 min. The absorbances were red at two wavelengths (550 and 690 nm). Cell viability in a well was defined as the percentage of the mean value of optical density resulting from the six cell controls, which was set 100% cell viability. The inhibitor concentration that reduces the mean value of optical density determined for cell controls by half is called 50% cytotoxic concentration (CC_50_). A minimum of three independent experiments were performed.

### Antiviral assays

Replication of the applied rhinoviruses and influenza viruses causes morphological changes and cell death (called cytopathic effect; CPE) of infected HeLa Ohio and MDCK cells, respectively. The CPE can be quantified after staining the cells with crystal violet and dye elution by optical density measurement as described before [34, 39]. The ability of compounds or extracts to protect cells from the virus-induced CPE can be analyzed using CPE inhibitory assays. Seeding and growth of MDCK cells was done similarly to the cytotoxicity assay with some modifications. Thus, HeLa Ohio cells were incubated for one day only. In addition, we washed the MDCK cells once with 100 μL test medium to remove the fetal calf serum hampering the influenza virus replication immediately before beginning with the CPE inhibitory assay.

CPE inhibitory assays started with the aspiration of the cell growth medium (rhinoviruses) or test medium (influenza viruses). Immediately thereafter, we added 50 μL of test medium (mock-treatment of cell and virus controls; each *n* = 6) or eight half-log dilutions of reference compounds or test items in test medium. Then we inoculated 50 μL of a virus suspension consisting a certain multiplicity of infection (MOI = TCID_50_ of virus used for infection in a well/number of cells present in that well) of the respective test virus in 50 μL of the test medium to the cell monolayers. The MOI of rhinovirus A2, rhinovirus B14, influenza virus A/Hong Kong/68, and influenza virus A/Jena/8178/09 was adjusted to 0.03, 0.01, 0.008, and 0.005, respectively. The concentration range of reference compounds (pleconaril or zanamivir) applied to the CPE inhibitory assays was 0.0003–1 μM. The concentration range of test items applied to test was 0.0003–0.5% v/v for thyme extract, 0.0316–100 μg/mL for pelargonium extract, and 0.0316–100 μM for ambroxol, bromhexine, and N-acetylcysteine. Six wells of non-infected and six wells of infected cells (both mock-treated with test medium) served as cell and virus control, respectively, on each plate.

Plates were incubated at 37 °C in a humidified atmosphere with 5% carbon dioxide for 48 h (influenza A viruses) or 72 h (rhinovirus A2), or at 33 °C for 72 h (rhinovirus B14) until a complete CPE was visible in the six mock-treated virus control wells under a light microscope. Thereafter, the viable cells were fixed and stained with the Dynex Immuno Assay System (Guernsey, UK). We described the details in the paragraph cytotoxicity determination. Crystal violet elution and optical density determination were also described there. The percentage of CPE inhibition by the test compounds was calculated according to Pauwels et al. [40] using the following equation: [(optical density measured with a given concentration of the test compound or extract in virus-infected cells – mean optical density of six virus controls) / (mean optical density of six cell controls - mean optical density of six virus controls)] × 100%. A 100% CPE inhibition means that the optical density of virus-infected, inhibitor-treated cells corresponded with the mean optical density of the six cell controls which was set 100% cell viability. Hence, 100% of virus-infected, inhibitor-treated cells were viable. At least three independent experiments were performed.

### Time-of-addition assays with pelargonium extract

We analyzed the inhibitory activity of pelargonium extract (50 μg/mL; triplicates) on different steps of the replication cycle of influenza virus A/Hong Kong/68 with plaque-reduction assays in MDCK cells grown in 12-well cell culture plates. We added pelargonium extract for different times and at different temperatures as described previously for another inhibitor [41]. Triplicates of mock-treated, not infected (cell control) and mock-treated, infected MDCK cells (virus control), and zanamivir-treated (1 μM; reference inhibitor), infected MDCK cells were included on each cell culture.

We compared the inhibitory effect of pelargonium extract and zanamivir using the following times and temperatures:

- Pre-treatment of cells. MDCK cells were treated with zanamivir or pelargonium extract for 1 h at 37 °C. Zanamivir and the extract were washed out before virus inoculation.
- Pre-treatment of virus. Virus suspension was incubated with zanamivir or pelargonium extract for 1 h at 37 °C and subsequently diluted to non-effective inhibitor concentrations (1:10,000) prior inoculation to MDCK cells.
- During adsorption. Zanamivir or pelargonium extract were added during virus adsorption at 4 °C for 2 h, a temperature and time allowing viruses to attach to MDCK cells but not to replicate.
- After adsorption: Virus was allowed to attach to MDCK cells as in c) but without zanamivir or pelargonium extract. After aspirating non-adsorbed virus, we added zanamivir or pelargonium extract with the agar-overlay to the virus-infected MDCK cells for 72 h.
- During adsorption till end. Virus attached to MDCK cells as in c). After washing away non-adsorbed virus, we added zanamivir or pelargonium extract with the agar-overlay to the virus-infected MDCK cells for 72 h.

At the end of the incubation time, we added a crystal violet solution (0.25% crystal violet (w/v), 0.5% ethanol, 4% formalin solution in water; 300 μL/well) for cell fixation, virus inactivation, and staining overnight. After removal of the agar overlay and washing of MDCK cell monolayers with tap water for removal of excess stain, we counted the plaques over a light box. We calculated the percentage of plaque production in zanamivir- or pelargonium extract-treated virus-infected MDCK cells by setting the mean number of plaques of the 3 virus controls to 100%.

### Hemagglutination and neuraminidase inhibition assay with human erythrocytes

We used a standard protocol developed for quantification of influenza virus-specific antibodies [42] to evaluate the effect of pelargonium extract on hemagglutination. Briefly, 25 μL of an influenza virus A/Jena/8178/09 suspension consisting 4 hemagglutination units was added to an equal volume of phosphate-buffered saline (hemagglutination control; *n* = 8), serial half-logarithmic dilutions of zanamivir (duplicates; maximum test concentration: 1 μM) or pelargonium extract (duplicates; maximum test concentration: 100 μg/mL) in a U-bottom 96-well polystyrene microplate. The negative control (no hemagglutination) consists only 50 μL phosphate-buffered saline (*n* = 8). We also tested the hemagglutination capacity of zanamivir or pelargonium extract dilutions in the absence of viral antigen to exclude unwanted effects e.g. erythrocyte lysis or induction of hemagglutination on the assay readout. We added 50 μL of a 1% human erythrocytes solution to each well of the microplate. After a two-hour incubation at 4 °C, we recorded the lowest compound concentration that induces hemagglutination or inhibits virus-mediated hemagglutination (minimum hemagglutination or hemagglutination inhibitory concentration, respectively).

Thereafter, we incubated the assays at 37 °C for further 24 h allowing viral neuraminidase activation [43]. The activated viral neuraminidase abrogates the virus-mediated hemagglutination in absence of inhibitors (hemagglutination control) or in the presence of ineffective inhibitor concentrations. Neuraminidase inhibitors prevent this abrogation of virus-mediated hemagglutination. We recorded the lowest compound concentration that inhibits the neuraminidase activity as the minimum neuraminidase inhibitory concentration and repeated the assay once for result confirmation.

### Data analysis

The 50% cytotoxic and inhibitory concentrations (CC_50_ and IC_50_ values) were calculated from dose-response curves. Linear regression analysis using Microsoft Excel 2010 was applied in the linear scaled dose-dependent sample concentrations. We calculated means and standard deviations of the calculated 50% cytotoxic and the 50% inhibitory concentrations using Microsoft Excel 2010.

## Results

### Cytotoxicity in HeLa Ohio cells and anti-rhinovirus activity

**Fig. 1:**
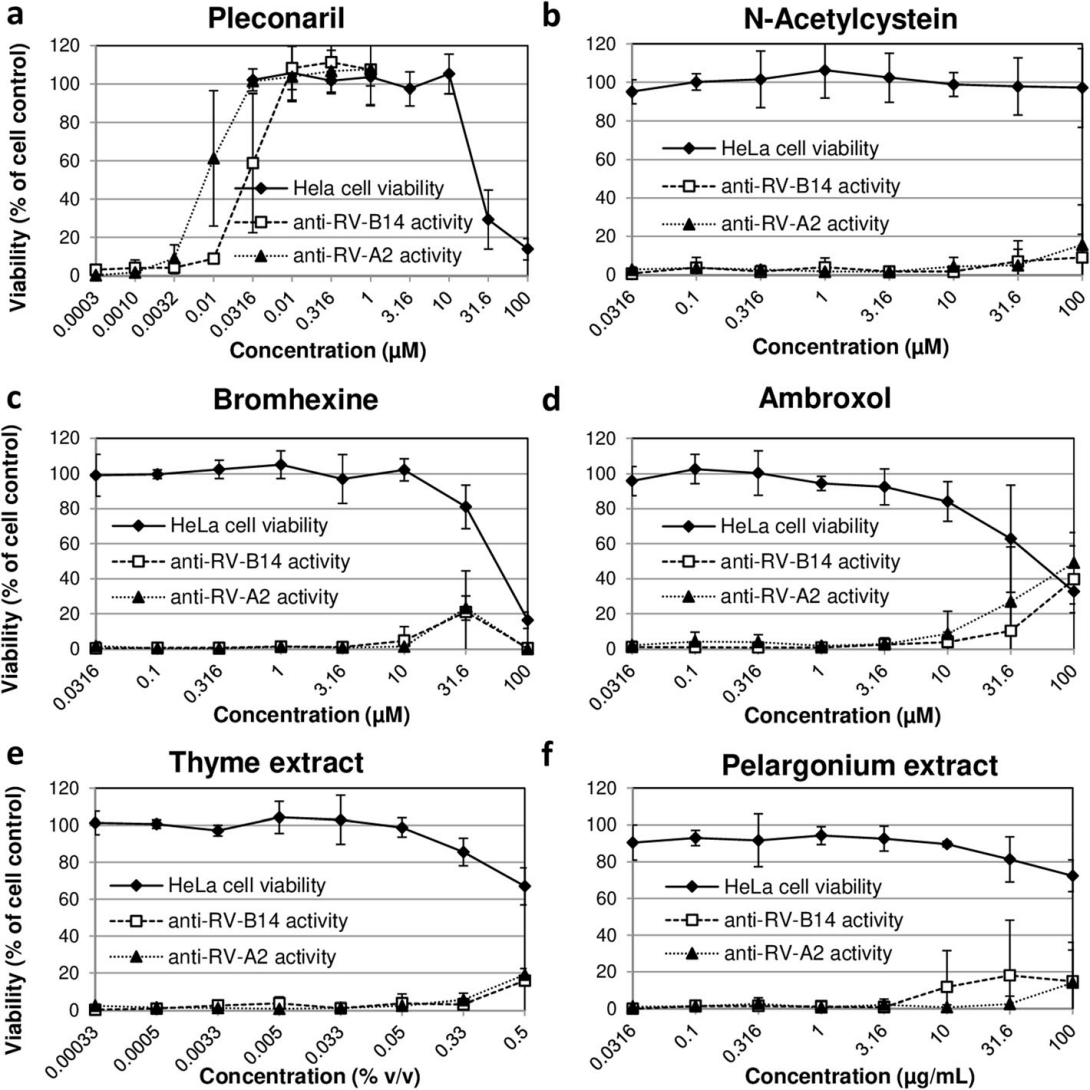
Dose-dependent cytotoxicity and anti-rhinovirus activity of pleconaril and test items in HeLa Ohio cells. The cytotoxicity and anti-influenza activity of zanamivir (**a**), N-acetylcystein (**b**), bromhexine (**c**), ambroxol (**d**), thyme extract (**e**), and pelargonium extract (**f**) was analyzed in MDCK cells. The percentage of cell viability was used as readout for analyzing the cytotoxic and antiviral effects. Cell viability was evaluated as the percentage of the mean value of optical density resulting from 6 cell controls, which was set 100%. Cytotoxic compounds have the potential to reduce cell viability. Influenza virus A/Hong Kong/68 (HK/68) and influenza virus A/Jena/8178/09 (Jena/8178) were included in the antiviral studies. Replication of both viruses results in a complete cytopathic effect of untreated MDCK cells thereby also reducing cell viability. Effective compound/extract treatment enhances the percentage of cell viability. Means and standard deviations of at least three independent experiments are shown

The cytotoxicity of test items for HeLa Ohio cells was quantified using a crystal violet staining procedure at day three after compound addition. Table 1 and Fig. 1 summarize the results. Only non-cytotoxic concentrations (≥90% viability as compared to the mean optical density value of six cell controls which was set 100% cell viability) were used to assess antiviral activity to avoid an impairment of virus growth due to cytotoxic effects of the test items.

**Table 1:** Cytotoxicity and antiviral activity of test items and control compounds

Compounds/Extracts	Units	50% Cytotoxic concentration in				50% Inhibitory concentration against								Selectivity index against	
						Rhinoviruses				Influenza A viruses				Influenza A viruses	
		HeLa cells		MDCK cells		A2		B14		Jena/8178		HK/68		Jena/8178	HK/68
		Mean	SD	Mean	SD	Mean	SD	Mean	SD	Mean	SD	Mean	SD		
**Pleconaril**	**μM**	28.75	8.06	n.d.		0.008	0.003	0.025	0.009	n.d.		n.d.			
**Zanamivir**	**μM**	n.d.		> 100^a^		n.d.		n.d.		0.007	0.004	0.008	0.003	> 14,285	> 12,500
**Bromhexine**	**μM**	51.85	9.80	51.29	9.08	n.a.		n.a.		n.a.		n.a.			
**Ambroxol**	**μM**	61.24	42.03	> 100^a^		n.a.		n.a.		n.a.		n.a.			
**N-acetylcysteine**	**μM**	> 100^a^		> 100^a^		n.a.		n.a.		n.a.		n.a.			
**Thyme extract**	**% v/v**	> 0.5^a^		0.32	0.07	n.a.		n.a.		0.03	0.01	0.03	0.00	11	11
**Pelargonium extract**	**μg/ml**	> 100^a^		> 100^a^		n.a.		n.a.		7.80	2.55	11.67	6.03	> 13	> 9

Means and standard deviation (SD) of the calculated 50% cytotoxic and the 50% inhibitory concentrations are shown

*n.d* not determined, *n.a* not active up to the maximum non-cytotoxic concentration

^a^more than 50% viability at the maximum tested concentration. Therefore, the 50% cytotoxic concentration is assumed to be higher than 100 μg/ml, 100 μM, or 0.5% v/v

The control compound pleconaril exerted a marked dose-dependent cytotoxic effect in HeLa Ohio cells when applied at concentrations higher than 10 μM (Fig. 1a). However, pleconaril dose-dependently inhibited the rhinovirus A2- and rhinovirus B14-induced CPE at low micromolar concentrations that were well tolerated by HeLa Ohio cells (Fig. 1a, Table 1). Thus, the CC_50_/IC_50_ ratio indicating the selectivity of pleconaril activity was > 3500.

With the exception of thyme extract, all test items were well tolerated in Hela Ohio cells. N-acetylcysteine was non-cytotoxic in the tested concentration range (Table 1). However, in contrast to pleconaril and as seen in Fig. 1b-f, none of the tested over-the-counter products reduced the rhinovirus A2- and rhinovirus B14-induced CPE at concentrations that are non-cytotoxic for HeLa Ohio cells. A slight inhibition of rhinovirus-induced CPE was seen at concentrations affecting also cell viability. Thus, we cannot exclude an unspecific inhibition of viral replication mediated by the measured cytotoxic effect.

### Cytotoxicity in MDCK cells and anti-influenza a virus activity

**Fig. 2:**
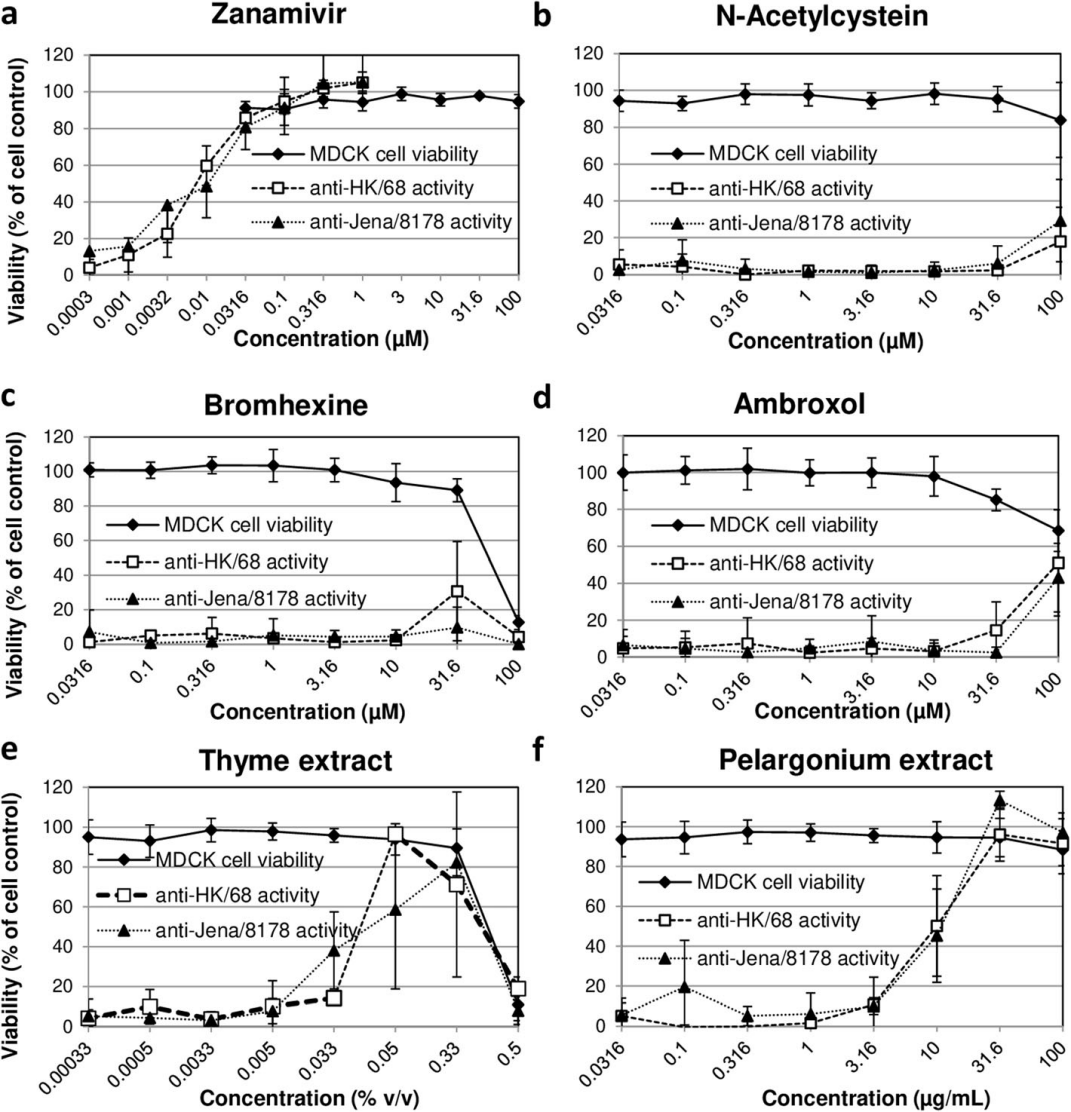
Dose-dependent cytotoxicity and anti-influenza virus activity of zanamivir and test items in MDCK cells. The cytotoxicity and anti-influenza virus activity of zanamivir (**a**), N-acetylcystein (**b**), bromhexine (**c**), ambroxol (**d**), thyme extract (**e**), and pelargonium extract (**f**) was analyzed in MDCK cells. The percentage of cell viability was used as readout for analyzing cytotoxic and antiviral effects. Cell viability was evaluated as the percentage of the mean value of optical density resulting from 6 cell controls, which was set 100%. Cytotoxic compounds have the potential to reduce cell viability. Influenza virus A/Hong Kong/68 (HK/68) and influenza virus A/Jena/8178/09 (Jena/8178) were included in the antiviral studies. Replication of both viruses results in a complete cytopathic effect of untreated MDCK cells thereby reducing cell viability. Effective compound/extract treatment enhances the percentage of cell viability. Means and standard deviations of at least three independent experiments are shown

The control compound zanamivir was well tolerated by MDCK cells (Fig. 2a). Zanamivir inhibited the influenza virus A/Hong Kong/68- and influenza virus A/Jena/8178/09-induced CPE at nano- and micromolar concentrations in a dose-dependent manner as expected (Fig. 2a, Table 1).

As seen in Table 1, Fig. 2b and d, N-acetylcysteine and ambroxol did not show any effect on influenza virus A/Hong Kong/68 and influenza virus A/Jena/8178/09 at non-cytotoxic concentrations (MDCK cell viability > 90%)..

Bromhexine was cytotoxic at 100 μM (Fig. 2c). At 31.6 μM the influenza virus A/Hong Kong/68-induced CPE was reduced by 30%.

Thyme extract was cytotoxic at the maximum tested concentration of 0.5% v/v (Fig. 2e). At lower, non-cytotoxic concentrations, the thyme extract dose-dependently decreased the influenza virus A/Hong Kong/68- and influenza virus A/Jena/8178/09-induced CPE indicating an antiviral activity.

The pelargonium extract exerted the strongest dose-dependent anti-influenza virus effect compared to the other 4 tested over-the-counter products. It showed IC_50_ values of approximately 10 μM against both, influenza virus A/Hong Kong/68- and influenza virus A/Jena/8178/09 and was non-cytotoxic at the tested concentrations (Table 1, Fig. 2f).

In summary, there was no antiviral activity against rhinovirus A2 and rhinovirus B14 at non-cytotoxic concentrations. Thyme and pelargonium extract inhibited the influenza virus-induced cytopathic effect in MDCK cells, whereas the other test items did not have antiviral activity.

### Treatment with pelargonium extract before, during, and after virus inoculation to MDCK cells inhibits plaque production of influenza virus A/Hong Kong/68

We performed time-of-addition assays to see the effect of pelargonium extract (50 μg/mL) on different steps of the viral life cycle. Therefore, we added pelargonium extract at different time points and temperatures before, during (adsorption), and after influenza virus A/Hong Kong/68 inoculation to MDCK cells in plaque-reduction assays. Zanamivir (1 μM) was included as reference.

**Fig. 3:**
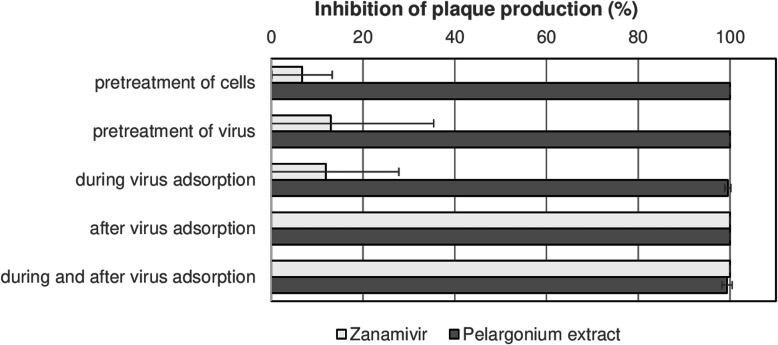
Pelargonium extract affects various steps of the replication cycle of influenza virus A/Hong Kong/68. We performed plaque reduction assays with influenza virus A/Hong Kong/68 and pelargonium extract (50 μg/mL) in MDCK cells. Zanamivir (1 μM) was included as reference inhibitor. We added pelargonium extract or zanamivir to MDCK cells and/or influenza virus A/Hong Kong/68 at different times before virus infection (pretreatment of cells or virus; at 37 °C for 1 h), during virus adsorption (at 4 °C for 2 h), after virus adsorption (at 37 °C for about 72 in the agar overlay), and during as well as after adsorption. After an incubation at 37 °C for 72 h and crystal violet staining, we counted the plaques over a light box. The mean plaque number of three mock-treated virus controls was set to 100%. The bars indicate the mean and standard deviation of the percent plaque reduction by pelargonium extract and zanamivir compared to the mock-treated virus control. Means and standard deviations of three independent experiments are shown

As expected, the reference compound zanamivir did not prevent the replication of influenza virus A/Hong Kong/68 when added before infection or during adsorption but completely blocked plaque production when added after viral adsorption as well as during and after viral adsorption (Fig. 3). In contrast, we observed a nearly complete inhibition of plaque production after treating MDCK cells or influenza virus A/Hong Kong/68 with pelargonium extract before infection (Fig. 3, pretreatment of cells or virus). Pelargonium extract also blocked the plaque production when treatment occurred during virus adsorption, after adsorption, and both during adsorption and after adsorption (Fig. 3). Thus, pelargonium extract blocked various steps of the viral life cycle.

### Pelargonium extract affects hemagglutination and inhibits the viral neuraminidase activity

To see whether pelargonium extract binding to the viral envelope or cell surface impairs the binding of the viral hemagglutinin to sialic acid-consisting receptors on the cells surface (adsorption) and/or the function of the neuraminidase, we performed hemagglutination assays with influenza virus A/8178/09 and human erythrocytes. The neuraminidase inhibitor zanamivir was included as control.

**Fig. 4:**
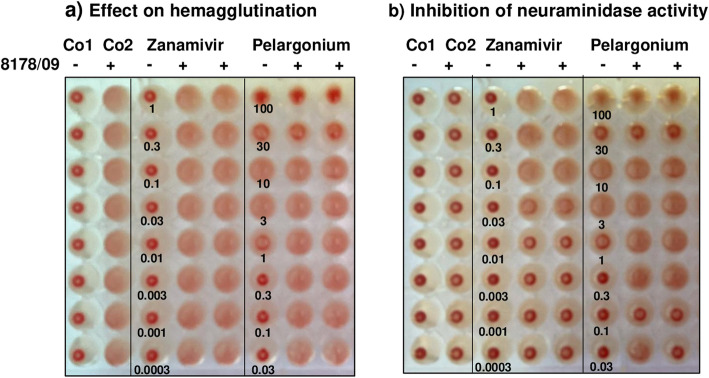
Representative photographs visualizing the effects of pelargonium extract on virus-mediated hemagglutination and viral neuraminidase activity. **a** Effect on hemagglutination: Four hemagglutination units of influenza virus A/Jena/8178/2009 (8178/09: +) or phosphate-buffered saline (8178/09: -) were mixed with phosphate-buffered saline or with serial half-logarithmic dilutions of zanamivir (1 to 0.0003 nM) or pelargonium extract (100 to 0.03 μg/mL). Then we added a human erythrocyte solution at 4° for 2 h. No hemagglutination occurs in the absence of virus in control 1 (Co1). Control 2 shows the virus-mediated hemagglutination of mock-treated human erythrocytes (Co2). Pelargonium extract induced a hemagglutination at 3 and 10 μg/mL and blocked the influenza virus-mediated hemagglutination at 30 and 100 μg/mL. **b** Inhibition of viral neuraminidase activity: After protocolling the effect on hemagglutination, we further incubated the test at 37 °C overnight. In the absence of inhibitors, the activated viral neuraminidase abolished the virus-mediated hemagglutination as seen in Co2. Both zanamivir and pelargonium extract blocked the neuraminidase activity with minimal inhibitory concentrations of 0.03 μM and 0.3 μg/mL, respectively. We repeated the experiments once to confirm the inhibitory effects

There was no hemagglutination in the absence of virus (Fig. 4a; Co1). Influenza virus A/8178/09 mediated the hemagglutination of the human erythrocytes (Fig. 4a; Co2). Incubation of pelargonium extract with human erythrocytes also caused hemagglutination at 3.16 and 10 μg/mL in the absence of influenza virus, (Fig. 4a). Higher concentrations of pelargonium extract prevented influenza virus-mediated hemagglutination (Fig. 4a). Zanamivir neither mediated nor inhibited hemagglutination as expected (Fig. 4a).

The neuraminidase cleaved the bounds between the viral hemagglutinin and the sialic acid bounds of the mock-treated hemagglutination control during incubation at 37 °C for 24 h causing abrogation of virus-mediated hemagglutination in control 2 (Fig. 4b; Co2). Both zanamivir and pelargonium extract treatment blocked the cleavage activity of the viral neuraminidase (Fig. 4b). Hence, abrogation of hemagglutination did not occur. The minimal neuraminidase inhibitory concentration of zanamivir and pelargonium extract was 0.03 μM and 0.3 μg/mL, respectively. In conclusion, pelargonium extract interfered with influenza virus binding to its receptors (adsorption) as well as neuraminidase activity crucial for viral release and transmission.

## Discussion

In the presents study, we compared the effect of 5 commonly used over-the-counter products for treatment of acute respiratory infections on the replication of 2 rhinoviruses and 2 influenza A viruses under standardized experimental conditions (e. g. MOI, compound treatment, spectrophotometric readout, control compounds) in an in vitro setting. The previously reported antiviral activity of the control compounds pleconaril [33, 34] and zanamivir [36, 37] was fully confirmed here. The results indicate a distinct anti-influenza A virus potential of thyme and pelargonium extracts.

At non-cytotoxic concentrations, neither bromhexine nor its metabolite ambroxol (0.0316–100 μM) exerted a ≥ 50% CPE inhibition against the tested rhinoviruses and influenza A viruses in HeLa Ohio and MDCK cells, respectively, when added immediately before virus challenge to the cells. Ambroxol reaches a mean peak human plasma concentration of about 0.2 μM [44], a concentration that was non-cytotoxic and did not act antiviral here. In contrast, a 3-day pretreatment of primary cultures of human tracheal epithelial cells with ambroxol at a concentration of at least 0.1 μM reduced the titer of rhinovirus B14 in the supernatant in a concentration-dependent manner [24]. According to Yamaya et al., a 3-day pretreatment of the cells with ambroxol indirectly affected the rhinovirus B14 infection by reducing the virus receptor expression, the number of acidic endosomes, and the fluorescence intensity of the acidic endosomes compared to untreated or vehicle-treated cells. The antiviral effect of ambroxol added immediately before virus inoculation was not studied by these authors. Therefore, it remains unclear whether the different study outcome is based on the different cells used, the pretreatment with ambroxol or both. To the best of our knowledge, there are no publications on the anti-influenza virus effect of ambroxol in vitro. In mouse airways influenza virus replication was indirectly suppressed by ambroxol-induced increase of the concentration of suppressors of viral replication, such as pulmonary surfactant, mucus protease inhibitor, and immunoglobulin A [25]. The absence of pulmonary surfactant, mucus protease inhibitor, and immunoglobulin A in vitro could explain the lack of anti-viral activity in in vitro experiments.

No antirhinoviral activity was found for the antioxidant molecule N-acetylcysteine (0.0316–100 μM) in the present study. There are no publications on the anti-rhinovirus effect of N-acetylcysteine in vitro. The negative results obtained for influenza A viruses seem to be in contradiction with previous findings obtained with influenza A viruses in A549 cells [27, 28]. However, this previously published anti-influenza virus activity was observed after treating A549 cells at much higher concentrations (10 and/or 15 mM), starting with a 1 h or 24 h pre-incubation period prior to virus infection. In striking contrast, the maximal plasma concentration following the application of 600 mg N-acetylcysteine is only about 16 μM [45]. There are two reports on the protective effects (reduction of lethality and/or body weight loss) of N-acetylcysteine (0.2 or 1 g/kg body weight per day) in models of influenza infection in mice [29, 46]. Both reports explain the observed protective effects by the antioxidant activity of N-acetylcysteine attenuating pulmonary inflammation, which does not play a role in in vitro experiments. A third report did not confirm the protective effect of N-acetylcysteine (1 g/kg body weight per day) on survival and the mean survival time in influenza A virus-infected mice [26].

The thyme extract was well tolerated in both HeLa and MDCK cells. It was inactive against rhinoviruses but, reduced the influenza A virus-induced cytopathic effect at non-cytotoxic dilutions (0.03–0.33% v/v) in a dose-dependent manner. Thyme extract is also known to inhibit herpes simplex virus infections in vitro [47, 48] by inactivating the infectivity of the virus [48].

Pelargonium extract treatment did not inhibit the induction of CPE by rhinoviruses A2 and B14 in HeLa Ohio cells. However, it was shown to reduce rhinovirus A16 infection of human primary bronchial epithelial cells by down-regulating cell membrane docking proteins and up-regulating host defense proteins, as described recently [31]. Pelargonium extract exerted the best anti-influenza A virus activity with mean IC_50_ values of 7.80 and 11.67 μg/mL for the two influenza A virus strains tested (H1N1: Jena/8178 and H3N2: HK/69). These results assort well with data published for Pelargonium sidoides extract EPs® 7630 and other influenza A virus strains of subtype H1N1 and H3N2 in MDCK cells elsewhere [30, 32]. The results of our time-of-addition assays show that the tested pelargonium extract interacts with the membrane of MDCK cells as well as with the influenza virus envelope blocking plaque production in MDCK cells. We also found an interaction between pelargonium extract and the membrane of human erythrocytes that mediated hemagglutination. Moreover, pelargonium extract impaired viral hemagglutination as well as neuraminidase activity as did EPs® 7630 [32]. Hence, pelargonium extract inhibits various steps of the influenza virus life cycle. Polyphenolic compounds, in particular oligomeric and polymeric proanthocyanidins based on gallocatechin and epigallocatechin moieties (prodelphinidins), represent the antiviral active compounds in EPs® 7630. The benefit of EPs® 7630 treatment was also demonstrated in a mouse model of influenza where the extract was administered by inhalation [32].

## Conclusions

The results obtained in the present in vitro study suggest that the inhibition of influenza virus replication by thyme and pelargonium extract may contribute to the beneficial effects of these plant extracts on symptoms of acute respiratory infections.

## Data Availability

The datasets used and/or analyzed during the current study are available from the corresponding author on reasonable request.

## Abbreviations

CC_50_: 50% cytotoxic concentration
CPE: Cytopathic effect
IC_50_: 50% inhibitory concentrations
MDCK: Madin Darby canine kidney
MOI: Multiplicity of infection
SD: Standard deviation
